# Epigenetic modifications associated with suicide and common mood and anxiety disorders: a systematic review of the literature

**DOI:** 10.1186/2045-5380-2-10

**Published:** 2012-06-14

**Authors:** Abdulrahman M El-Sayed, Michelle R Haloossim, Sandro Galea, Karestan C Koenen

**Affiliations:** 1 Department of Epidemiology, Mailman School of Public Health, Columbia University, 722 W. 168th Street, R521, New York, NY 10032, USA; 2 College of Physicians and Surgeons, Columbia University, New York, NY, USA; 3 Department of Sociomedical Sciences, Mailman School of Public Health, Columbia University, 722 W. 168th Street, New York, NY, 10032, USA; 4 Department of Epidemiology, Mailman School of Public Health, Columbia University, 722 W. 168th Street, 1508, New York, NY, 10032, USA; 5 Department of Epidemiology, Mailman School of Public Health, Columbia University, 722 W. 168th Street, R720G, New York, NY, 10032, USA

**Keywords:** Epigenetics, Mood disorders, Anxiety disorders, Suicide, Depression, PTSD, Histone modification, Methylation

## Abstract

Epigenetic modifications are those reversible, mitotically heritable alterations in genomic expression that occur independent of changes in gene sequence. Epigenetic studies have the potential to improve our understanding of the etiology of mood and anxiety disorders and suicide by bridging the gap in knowledge between the exogenous environmental exposures and pathophysiology that produce common mood and anxiety disorders and suicide. We systematically reviewed the English-language peer-reviewed literature about epigenetic regulation in these disorders between 2001–2011, summarizing and synthesizing this literature with respect to directions for future work. Twenty-one articles met our inclusion criteria. Twelve studies were concerned with epigenetic changes among suicide completers; other studies considered epigenetic regulation in depression, post-traumatic stress disorder, and panic disorder. Several studies focused on epigenetic regulation of amine, glucocorticoid, and serotonin metabolism in the production of common mood and anxiety disorders and suicide. The literature is nascent and has yet to reach consensus about the roles of particular epigenetic modifications in the etiology of these outcomes. Future studies require larger sample sizes and measurements of environmental exposures antecedent to epigenetic modification. Further work is also needed to clarify the link between epigenetic modifications in the brain and peripheral tissues and to establish ‘gold standard’ epigenetic assays.

## Introduction

Nearly 50% of adults in the United States have experienced a mood or anxiety disorder at some point in their lives. Mood and anxiety disorders are among the most debilitating diseases worldwide—the World Health Organization’s latest Global Burden of Disease report ranked depression among the top three most prevalent causes of disability globally, accounting for the highest single proportion of years lived with disability around the world [1,2]. Panic disorder, as well as self-inflicted injury, which is often precipitated by depression, were also high on the WHO list [2]. Mood, anxiety disorders and self-inflicted injury are also profoundly expensive, imposing high direct and indirect costs on individuals, industry, and health systems, alike [3].

The search for causes of common mood and anxiety disorders and suicide has spanned at least a century of research in a wide range of disciplines. However, a gap remains between studies focused on exogenous environmental determinants such as negative life events [4] or neighborhood social context [5,6] and studies focused on genetic determinants and biological correlates such as abnormalities in brain circuitry [7]. The recent growth in interest in epigenetic studies in human populations has been fueled, in part, by the potential of epigenetics to bridge this divide [8].

Epigenetic modifications are those reversible, mitotically heritable alterations in genomic expression that occur independent of changes in gene sequence [9]. Rather they occur via methylation of DNA or alterations to chromatin structure that either impede or facilitate access to the DNA by transcription factors and associated complexes [9]. Epigenetic modification of expression has been demonstrated to mediate the interplay between environmental stimuli and physiologic—and pathophysiologic—change throughout the life course [10]. Early research has been promising [11], demonstrating epigenetic involvement in the pathophysiology of depression [12], anxiety disorders [13], and suicide [14]. However, a central challenge to understanding the role of epigenetic modifications in psychopathology is limited access to brain tissue in extant studies, where inference has been limited to assessments of epigenetic modification of peripheral tissue samples which may not be related to pathophysiologic processes underlying psychopathology.

Given the growing interest in behavioral epigenetics it appears to be the appropriate time to take stock, and to synthesize the peer-reviewed literature about epigenetic modification in the etiology of common mood and anxiety disorder and suicide. In reviewing the existing literature, we aimed to summarize the state of the science, identify methodological challenges, and offer potential solutions to address these challenges.

## Methods

We systematically reviewed the literature about epigenetic factors in the etiology of mood and anxiety disorders and suicide. We restricted our search to English-language, peer-reviewed articles. Our review encompassed the literature published between January 1^st^, 2001 and December 1^st^, 2011—we limited our review to the years following the sequencing of the human genome to reflect current thinking about the genetics of psychopathology. The literature reviewed here was identified via the MEDLINE and PSYCHINFO databases.

Our original search yielded 1273 articles, 600 of which were judged to consider epigenetic factors in the etiology of mood and anxiety disorders and/or suicide after screening by title. Another 368 were discarded after screening by abstract because they did not consider epigenetic mechanisms or disease outcomes of interest. Of the remaining 232, articles were included in the review if they fulfilled the following criteria:

· Included original data about a n > 1 sample of human subjects

· Used DSM-III, DSM-IV, or ICD-10 criteria to classify participants as having common mood or anxiety disorders (including and limited to PTSD, GAD, Phobias, Panic Disorder, or Depression) OR used coroners’ reports to classify cause of death by self-inflicted injury

· Objectively assessed epigenetic mechanisms relating to the etiology of mood and anxiety disorders or suicide

**Figure 1:**
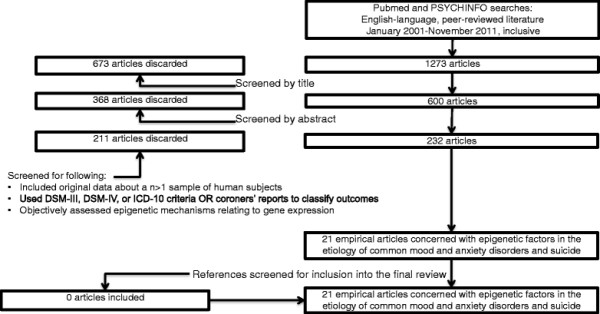
Review search strategy: Epigenetic modification in the etiology of common mood and anxiety disorders and suicide.

After reading the complete manuscripts, 211 were excluded because they did not meet our criteria, above. This left 21 articles from the original search considered in this review. Reference lists from these articles were searched, and yielded no further articles which fulfilled the inclusion criteria, yielding a final total of 21 articles reviewed here. Figure1 shows a flow diagram of our search strategy.

Because of the diversity of outcomes in which we were interested, the genetic pathways considered in the extant literature, and the methods used to both assess for epigenetic mechanisms and statistically analyze the findings, a meta-analysis of the results would not have been appropriate or feasible. For each of the 21 papers, we extracted the following information: the outcome of interest; sample population; proportion male; proportion White; number of cases and controls; loci considered; tissues sampled; laboratory techniques used; statistical analyses employed; and summary of the findings.

## Results

Table 1 includes a detailed review of each of the studies included in our review. The majority (12) of the studies included in the literature considered epigenetic modification in the etiology of suicide [14-25]. Five studies considered epigenetic factors and mood disorders (all five were concerned with depression) [12,26-29], and the remaining four studies considered epigenetic factors in the etiology of anxiety disorders (three considered PTSD and one considered panic disorder) [13,30-32]. 

**Table 1:** *Studies about epigenetic modifications involved in the etiology of common mood and anxiety disorders and suicide, January 2010-November 2011

**Author (Year)**	**Outcome**	**Sample**	**% Male**	**% White**	**N Cases**	**N Controls**	**Gene (s)/loci**	**Tissue**	**Assay**	**Statistical analysis**	**Summary**
**Suicide**											
Poulter et al., 2008 [15]	Completed suicide	Department of Forensic Medicine, Semmelweis University Medical School, Budapest	50% in methylation analyses	100%	10 for methylation analysis	10 for methylation analysis	DNMT mRNA expression in the frontopolar cortex, hippocampus, amygdala, and dorsal vagal complex; GABAa promoter	post-mortem brain	qPCR; Methylation Mapping; Western Blot	ANOVA and *t*-tests with Bonferroni corrections; chi-square tests	DNMT-1 was downregulated, and DNMT-3B was upregulated among suicide completers relative to controls in the frontopolar cortex. There was no association between DNMT expression and suicide in the amygdala. In the hippocampus, DNMT-1 and 3B were downregulated in suicide completers relative to controls. DNMT-3B was elevated in the hypothalamus and the dorsal vagal complex. CG2 and 4 were hypermethylated in suicide completers relative to controls in the GABAa alpha1 subunit, and this was associated with DNMT-3b upregulation.
McGowan et al., 2008 [14]	Completed suicide following child abuse	Quebec Suicide Brain Bank matched on age, gender, and post-mortem interval; 5 cases and 1 control used for RNA expression analyses, separate 13 cases and 11 controls used for other analyses	100%	100%	18	12	rRNA promotor in the hippocampus and cerebellum	post-mortem brain	RT-PCR; methylation mapping; nearest neighbor quantification	factorial ANOVA, Bonferroni corrections; linear regression for site-specific differences	rRNA promotor was more heavily methylated in the hippocampus of suicide subjects than of controls. 21 of 26 sites had higher methylation frequency in suicide subjects relative to controls in the hippocampus. These differences were not observed in the cerebellum.
Guipponi et al., 2008 [16]	Completed suicide	Geneva, Switzerland Institute for Forensic Medicine	55% cases; 50% controls	N/I	20	20	SSAT promotor in ventral prefrontal cortex	post-mortem brain	Pyrosequencing	ANOVA and *t*-tests	There was no association between promotor methylation and suicide.
De Luca et al., 2009 [17]	Completed suicide	Stanley Medical Research Institute array collection; suicide and non-suicide controls matched on genotype, diagnosis, age at death, and sex	40% cases; 60% controls	N/I	10	10	5-HT2A C102 allele in the dorsolateral pre-frontal cortex	post-mortem brain	Hpall treatment followed by TaqMan Assay Q-PCR	independent *t*-tests	No significant difference in methylation levels of C102 were found in Brain-tissue between suicide completers and controls.
	Suicide attempt	48 schizophrenics (24 suicide attempts and 24 non-attempters) pooled from Toronto Schizophrenia, and SMR populations	67% cases; 75% controls	N/I	24	24	5-HT2A C102 allele in the white blood cells	leukocytes	Hpall treatment followed by TaqMan Assay Q-PCR	independent *t*-tests, corrected by Bonferroni	Suicide attempters had significantly higher methylation of 102 C in peripheral leukocytes relative to controls.
	Suicide attempt	57 bipolar subjects (29 suicide attempts and 28 non-attempters) pooled from Toronto Bipolar and SMR populations	34.4% cases; 35.7% controls	N/I	29	28	5-HT2A C102 allele in the white blood cells	leukocytes	Hpall treatment followed by TaqMan Assay Q-PCR	independent *t*-tests, corrected by Bonferroni	There was no significant difference in methylation of 102 C in the peripheral leukocytes of suicide attempters and controls.
Ernst et al., 2009 [18]	Completed suicide	Quebec Suicide Brain Bank (28 suicide completers and 11 controls); matched on age, postermortem interval, and pH. Epigenetic analyses performed on 10 subjects with low TrKB expression and 10 matched controls.	100%	100%	10 (of 28 possible)	10 (of 11 possible)	TrKB.T1 promotor region in the frontal cortex	post-mortem brain	Methylation mapping; Q-PCR and Western blot analysis; HG-U133 plus 2 microarray chip	*t*-test on mean methylation at two CpG dinucleotides	There was a significant difference in TrKB.T1 expression among suicide completers and non-completers. In suicide completers, downregulation was correlated with methylation frequency at sites 2 and 5 in the TrKB.T1 promotor.
McGowan et al., 2009 [19]	Completed suicide following child abuse	Quebec Suicide Brain Bank matched on age, gender, and post-mortem interval	100%	100%	24 (12 with history of child abuse; 12 without such a history)	12	NR3C1 neuron-specific glucocorticoid receptor promotor in the hippocampus	post-mortem brain	RT-PCR; methylation mapping	factorial ANOVA, Bonferroni corrections	There was decreased glucocorticoid receptor mRNA and increased cytosine methylation in abused suicide completers as compared to non-abused completers. There were no differences between non-abused completers and non- completers.
Klempan et al., 2009 [20]	Completed suicide among depressive patients	Quebec Suicide Brain Bank matched on age	100%	100%	16 (4 included in methylation analysis)	13 (4 included in methylation analysis)	QKI promotor in the orbitofrontal cortex	post-mortem brain	HG-U133 A/B microarray; methylation mapping; TaqMan gene expression assays	*t*-test	Suicide completers had significantly lower mRNA levels of QKI in 11 cortical regions and in the amygdala. However, there were no significant differences in methylation levels at the QKI promotor by suicide status.
Keller et al., 2010 [22]	Completed suicide	Biological Bank of the Institut za Varovanje Zdravja, Ljubljana (Slovenia)	48% cases; 48% controls	100%	44	33	Overall methylation in Wernicke's area; BDNF promotor region methylation in Wernicke's area	post-mortem brain	Pyrosequencing; MassARRAY; bisulfite genomic sequencing	ANOVA and ANCOVA adjusted for sex and age	BDNF promotor IV methylation was significantly higher in suicide completers relative to controls. Higher BDNF methylation was associated with lower BDNF transcript IV. There was no difference in genome-wide methylation levels between suicide completers and controls. In suicide completers with high methylation levels, there were lower BDNF mRNA levels.
Fiori et al., 2010 [25]	Completed suicide	Quebec Suicide Brain Bank as well as a Quebecois sample of healthy controls	100%	100%	10 (of 40 possible)	10 (of 56 possible)	SMS and SMOX promotor region in the BA 8/9, as well as histone methylation of H3K27me3	post-mortem brain	Methylation mapping and chromatin immunoprecipitation	*t*-test with correction for mutliple testing	There were no associations between methylation at any locus and suicide. Histone H3k27me3 methylation was not associated with suicide.
Fiori et al., 2011a [24]	Completed suicide	Quebec Suicide Brain Bank as well as a Quebecois sample of healthy controls	100%	100%	10	10	SAT1 promotor in the prefrontal cortex; histone methylation of H3K27me3	post-mortem brain	Methylation mapping and chromatin immunoprecipitation	*t*-test	CpG methylation at the SAT1 promotor was not associated with suicide, although it did predict decreased SAT1 expression. Histone H3k27me3 methylation was not associated with suicide completion.
Fiori et al., 2011b [23]	Completed suicide	Quebec Suicide Brain Bank as well as a Quebecois sample of healthy controls; suicide completers selected on over-expression of AMD1, ARG2, OAZ1, and OAZ2	100%	100%	34	34	Histone H3Kme3 in the inferior frontal gyrus	post-mortem brain	Chromatin immunoprecipitation	*t*-test and pearson correlation	Suicide completers had significantly higher H3Kme3 levels at OAZ1. H3Kme3 was positively correlated with expression of ARG2 and OAZ1.
Keller et al., 2011 [21]	Completed suicide	Biological Bank of the Institut za Varovanje Zdravja, Ljubljana (Slovenia)	61% cases; 52% controls	N/I	18	18	BDNF receptor (TrkB) in Wernicke's area; BDNF promotor in Wernicke's area	post-mortem brain	MassArray	*t*-tests	There were no significant differences in TrkB promotor methylation between suicide completers and controls. BDNF promotor IV methylation was significantly higher in suicide completers relative to controls.
**Mood Disorders**											
Philibert et al., 2008 [26]	Major depression	Iowa Adoption Study participants	50%	93%	68 (history of MD); 17 (current MD)	124 (no history of MD); 175 (no current MD)	5HTT promotor SLC6A4	lymphoblast	RT-PCR; MassArray quantitative methylation	ANOVA and linear regression with Bonferroni corrections	There was no relationship between methylation and mRNA expression overall. There was no relationship between SLC6A4 expression and life history of MD or current MD.
Alt et al., 2009 [27]	Major Depressive Disorder	Dutch Brain Bank; Matched on age, sex, brain weight, post-mortem delay and pH of CSF	67% cases; 50% controls	N/I	6	6	Glucocorticoid receptor promotor region (1 J, 1E, 1B, 1 F) of amygdala, hippocampus, inferior postulate gyrus, cingulate gyrus, nucleus accumbens	post-mortem brain	QIAamp DNA Mni kit (Qiagen); pyrosequencing	Mann–Whitney U-test, corrected by Bonferroni	GR transcript levels were homogenous by disease status. Exon 1 F expression was reduced among MDD patients relative to controls. There were no significant differences in methylation patterns between groups between different brain regions.
Olsson et al., 2010 [28]	Depression	Nested cohort from the Victorian Adolescent Health Cohort Study, a population-representative sample of 2032 young Australians in Victoria	55%	96%	25	125	5HTT promotor	buccal cells	MassArrray	logistic regression, Bonferroni corrections	Buccal cell 5HTT methylation and depression were not associated either over the entire promotor or in subregions identified by PCA. However, there was a joint effect of 5HTT methylation and the s-allele variant on risk for depression.
Fuchikami et al., 2011 [29]	Major depression	Japanese sample of DSM-IV criteria depressed patients and health controls from four academic medical centers	N/I	N/I	20	18	BDNF promotor	white blood cells	MassArray	2-dimensional hierarchical clustering and *t*-tests	Mean methylation rates of CpG 1 but not 4 at the BDNF promotor was associated with depression.
Uddin et al., 2011 [12]	Lifetime Depression	Detroit Neighborhood Health Survey, a multiethnic representative survey of low- income neighborhoods in Detroit, MI	40%	14%	33	67	non-specific	whole blood	HumanMethylation 27 beadchip; pyrosequencing of two loci	McNemar's chi-squared tests for overall methylation; Functional annotation clustering analyzed via Wilcoxon test, alpha < 0.01	Cases had fewer uniquely unmethylated and methylated genes than controls. Methylated genes were associated with lower gene expression. FACs associated with multicellular organismal development, lipoprotein activity, and hydrolase activity were uniquely unmethylated, while those associated with protease activity, metabolic processes, and cell development were uniquely methylated in cases. In controls, FACs associated with brain development, tryptophan metabolism, and neuormuscular processes were uniquely unmethylated, while those involved in signaling, lipocalin, and tissue development were uniquely methylated.
**Anxiety Disorders**											
Elser et al., 2006 [13]	Panic disorder	N/I	N/I	N/I	24	N/I	NET promotor and exon 9	white blood cells	CpGenome DNA modification it and ABI Prism 7700 Sequence Detection System; chromatin immunoprecipitation	*t*-test on mean methylation in promotor region	There was a significant difference in NET promotor methylation among patients with panic disorder relative to healthy controls. Promotor regions were also enriched with the MeCP2 co-repressor complex.
Uddin et al., 2010 [30]	Post-traumatic stress disorder	Detroit Neighborhood Health Survey, a multiethnic representative survey of low-income neighborhoods in Detroit, MI	40%	14%	27	77	non-specific	whole blood	HumanMethylation 27 beadchip	McNemar's chi-squared tests for overall methylation; Functional annotation clustering analyzed via Wilcoxon test, alpha < 0.01	There was no difference in overall methylation level among PTSD cases relative to controls, however the number of uniquely methylated genes did differ by disease status. Uniquely unmethylated genes in PTSD cases were associated immune system involvement, including TLR1, TLR3 (innate immune system), IL8, LTA, and KLRG-1 (adaptive immune system).
Uddin et al., 2011 [31]	Post-traumatic stress disorder	Detroit Neighborhood Health Survey, a multiethnic representative survey of low-income neighborhoods in Detroit, MI	40%	14%	27	77	33 genes previously described in the literature as associated with PTSD	whole blood	HumanMethylation 27 beadchip	Logistic regression to assess the relation between site-specific methylation and lifetime traumatic events adjusted for race, smoking, gender, age, socioeconomic status, peripheral cell count, and medication	Only MAN2C1 methylation interacted with number of potentially traumatic events to significantly predict lifetime PTSD. Increases in both factors were associated with increased lifetime PTSD risk.
Smith et al., 2011 [32]	Lifetime post- traumatic stress disorder	Cohort of African-American pariticipants recruited at clinical waiting rooms in a low-income, urban context	63% of cases; 60% of controls	0%	51 (25 with childhood trauma)	53 (21 with childhood trauma)	non-specific	whole blood	HumanMethylation 27 beadchip	linear mixed model adjusted for age, sex, and chip effects, with adjustment for multiple testing using the false discovery rate method	Lifetime PTSD was associated with increased methylation overall. Lifetime PTSD was associated with increased methylation in TPR, ANXA2, CLEC9A, ACPT5, and TLR8 compared to controls. CPG site methylation at BDNF and CXCL1 were associated with lifetime PTSD. There was no association between methylation at NR3C1 and SLC6A4 and PTSD.

*N/I; not indicated.

All of the studies concerned with epigenetic factors in suicide assessed gene expression and methylation profiles in the post-mortem brains of suicide completers relative to non-suicide controls [14-25], although one study also included adjuvant data about methylation profiles in peripheral leukocytes [17]. Among studies regarding epigenetic factors in the etiology of depression, one study assessed methylation and expression profiles in the post-mortem brain [27], another assessed methylation and expression of tissue in buccal cells [28], and the remaining three assessed methylation and expression profiles in peripheral blood [12,26,29]. All four studies about epigenetic mechanisms in anxiety disorders assessed methylation and expression in whole blood [13,30-32].

### Epigenetic modifications in the etiology of suicide

There were twelve studies concerned with epigenetic mechanisms in the etiology of suicide [14-25]. Three studies assessed epigenetic mechanisms involved in the expression of Brain Derived Neurotrophic Factor (BDNF) and its receptor, Tropomyosin-Related Kinase B (TrkB). In a study of post-mortem brain tissue from 10 suicide completers and 10 controls matched on age, gender, post-mortem interval and brain pH, Ernst and colleagues [18] found significantly higher methylation of the TrkB.T1 promoter in the frontal cortices of suicide completers relative to controls, and that methylation frequency at sites 2 and 5 of the promoter were associated with lower TrkB.T1 expression. By contrast, Keller and colleagues [22] demonstrated that no significant differences in TrkB.T1 methylation in Wernecke’s areas of suicide completers relative to non-suicide controls. However, in a 2010 study from the same sample, the group demonstrated that suicide completers had higher rates of methylation at BDNF promoter IV than non-suicide controls, and that BDNF promoter IV methylation was predictive of lower BDNF mRNA expression in cases relative to controls [21].

Several studies considered epigenetic modification of genes involved in amine metabolism in the brain. Three studies using data from the Quebec Suicide Brain Bank and non-suicide controls from the same area considered the role of histone methylation at different locations in the etiology of suicide with conflicting results. The first found no association between H3K4me3 methylation at either spermine synthase (SMS) or spermine oxidase (SMOX) and suicide [25]. In addition, there was no association between methylation at either site and risk of suicide [25]. Another found higher H3K4me3 methylation levels, a marker of more open chromatin, at the antizyme 1 (OAZ1) promoter [23]. H3K4me3 methylation was correlated with higher expression of OAZ1 and arginase II (ARG2). A third study by the same group also concerned with epigenetic modification of genes involved in amine metabolism found that spermidine/spermine N^1^-acetyltransferase (SAT1) methylation was not associated with suicide. Similarly, there was no association between H3K27me3 methylation and suicide. A fourth study using post-mortem brain samples of 20 suicide cases and 20 non-suicide controls from the Geneva Institute for Forensic Medicine considered methylation of the spermine/spermidine N^1^-acetyltransferase (SSAT) gene promoter in the ventral prefrontal cortex in the etiology of suicide and found no association between methylation and suicide.

One study was concerned with epigenetic modifications of genes involved in 5-HT metabolism. De Luca and colleagues studied both the post-mortem brains of suicide completers as well as peripheral blood methylation expression profiles of suicide attempters to assess the role of epigenetic modification of the 5-HT2A C102 in the etiology of suicide [17]. Comparing 10 suicide completers to non-suicide controls matched on genotype, age at death, and previous psychiatric diagnoses, they found no difference in methylation at C102 in the dorsolateral pre-frontal cortex [17]. They also compared methylation profiles at this location in the leukocytes of both suicide attempters and non-suicide controls all of whom had a history of bipolar disorder or schizophrenia. While there were no differences in methylation among bipolar patients, suicide attempters with schizophrenia had significantly higher methylation levels at the C102 location [17].

Poulter and colleagues used post-mortem brain samples from several brain regions from 10 suicide completers and 10 non-suicide controls in Budapest to assess the role of DNA methyltransferase (DNMT) expression and subsequent GABA_A_ promoter hypermethylation [15]. They found differential DNMT expression in the frontopolar cortices, dorsal vagal complexes, and hippocampi of suicide completers relative to controls, and no difference in the amygdala. Moreover, DNMT-3B upregulation predicted hypermethylation of CpG islands 2 and 4 in the GABA_A_ promoter [15].

One study considered the role of epigenetic regulation of the neuron-specific glucocorticoid receptor (NR3C1) in suicide etiology among those with a history of childhood abuse [19]. McGowan and colleagues found that in the hippocampi of post-mortem brain tissue among patients matched on age, gender and post-mortem interval, suicide completers with a history of child abuse had higher rates of CpG methylation and lower expression of NR3C1 mRNA than non-abused suicide completers [19]. There were no differences between non-abused suicide completers and non-suicide controls [19]. Another study by the same group also considered the role of epigenetic modification in the etiology of suicide among those abused during childhood [14]. They found more heavy methylation of the rRNA promoter in the hippocampi of suicide completers relative to non-suicide controls [14]. There was, however, no difference in methylation of the rRNA promoter in the cerebellum of suicide completers relative to controls [14].

A final study considered the etiology of suicide among depressive patients. Klempan and colleagues assessed differences in methylation and expression of the oligodendrocyte-specific RNA binding protein (QKI) in suicide completers with a history of major depression relative to age-matched non-suicide controls [20]. Although they found that suicide completers had significantly lower mRNA levels of QKI in 11 cortical regions as well as in the amygdala, there were no significant differences in methylation levels between suicide completers and non-suicide controls [20].

### Epigenetic modifications in the etiology of mood disorders

There were five studies concerned with the role of epigenetic modification in the etiology of mood disorders—all five were concerned specifically with depression [12,26-29]. Two studies were concerned with epigenetic influences in genes involved in 5-HT metabolism and depression with mixed results [26,28]. In a study of participants in the Iowa Adoption Study, Philibert and colleagues assessed the relationship between methylation at the 5-HT transporter (SLC6A4) in lymphoblasts and both history of and current major depression—they found no association between SLC6A4 methylation or mRNA expression and either outcome [26]. Another study assessed the relation between 5-HT transporter promoter methylation in buccal cells and depression in a nested cohort from the Victorian Adolescent Health Study in Victoria, Australia. While 5-HT transporter promoter methylation did not predict depression on its own, there was a significant interaction between methylation and the short “s-type” allele in predicting depression.

A study by Alt and colleagues assessed the relationship between glucocorticoid receptor (GR) promoter methylation in several regions of the post-mortem brains of depressed patients relative to non-depressed controls from the Dutch Brain Bank matched on age, sex, brain weight, post-mortem delay, and pH of the cerebrospinal fluid [27]. There were no differences in GR mRNA expression levels by disease status nor in methylation patterns in any of the brain regions sampled (amygdala, hippocampus, inferior postulate gyrus, cingulate gyrus, and nucleus accumbens) [27].

In a study of a Japanese sample of depressed patients and non-depressed controls recruited from four academic medical centers, Fuchikami and colleagues assessed the relation between BDNF promoter methylation in white blood cells and depression [29]. They found that BDNF promoter methylation at CpG site 1 was associated with depression, although there was no association between CpG methylation at site 4 and the outcome [29].

A final study considered “functional annotation clusters” (FACs) of epigenetic modifications in the whole blood of a community-based sample in Detroit, MI [12]. The authors found that cases had fewer uniquely unmethylated and methylated genes than controls, and that methylation predicted lower gene expression. FACs associated with multicellular organismal development, lipoprotein activity, and hydrolase activity were uniquely unmethylated, while those associated with protease activity, metabolic processes, and cell development were uniquely methylated among depressed subjects [12]. In controls, FACs involved in brain development, tryptophan metabolism, and neuromuscular processes were uniquely unmethylated, while those involved in signaling, lipocalin, and tissue development were uniquely methylated [12].

### Epigenetic modifications in the etiology of anxiety disorders

There were four studies that considered epigenetic mechanisms in the etiology of anxiety disorders [13,30-32]. Three studies were concerned with the etiology of post-traumatic stress disorder (PTSD) [30-32] and one was concerned with panic disorder [13].

With respect to PTSD, none of the three studies considered epigenetic modification of one specific gene pathway. Rather each considered non-specific sets of pathways. A study by Smith and colleagues of a cohort of African-American participants recruited at clinical waiting rooms in a low income context used linear mixed models adjusted for age, sex, and assay effects, found that increased methylation in white blood cells was associated with lifetime PTSD [32]. Moreover, those with lifetime PTSD had increased methylation at several sites, including translocated promoter region (TPR), annexin 2 (ANXA2), c-type lectin-like receptor 9A (CLEC9A), testicular acid phosphatase 5 (ACPT5) and toll-like receptor 8 (TLR8). Methylation at specific CpG islands in BDNF and chemokine ligand 1 (CXCL1) were also associated with lifetime PTSD [32]. Another study by Uddin and colleagues considered unique methylation and unmethylation by FAC [30] in white blood cells of a community-based sample in Detroit, MI. There was no difference in overall methylation levels among PTSD cases relative to controls, however the number of uniquely methylated genes did differ among cases and controls [30]. Uniquely unmethylated genes in PTSD cases were associated immune system involvement, including toll-like receptor 1 (TLR1), toll-like receptor 3 (TLR3) (innate immune system), interleukin 8 (IL8), lymphotoxin alpha (LTA), and killer cell lectin-like receptor G1 (KLRG-1) (adaptive immune system) [30]. Another study among the same population demonstrated effect modification of the relationship between the number of reported potentially traumatic events and the degree of mannosidase 2 C1 (MAN2C1) methylation, such that among those with increased methylation at MAN2C1 a larger number of potentially traumatic events was more strongly related to PTSD risk [31].

A fourth study was concerned with the role of epigenetic modification of the promoter as well as exon 9 of the neuroepithelial cell transforming (NET) gene promotor and exon 9 in the etiology of panic disorder [13]. Using peripheral blood, the authors demonstrated a significant difference in NET promotor methylation relative to healthy controls. Moreover, they noted an enrichment of the MeCP2 co-repressor complex at the promoter regions of NET among cases relative to controls [13].

## Discussion

We systematically reviewed the peer-reviewed literature about the role of epigenetic modification in the etiology of common mood and anxiety disorders and suicide. Twenty-one papers were published between 2001, the publication of the human genome project, and 2011. The majority (12) of studies we found were concerned with evidence of epigenetic changes in the post-mortem brains of suicide completers, with other studies considering epigenetic factors in the etiology of depression, PTSD, and panic disorder. A plurality focused on epigenetic regulation of genes involved in amine, glucocorticoid, and serotonin metabolism in the production of common mood and anxiety disorders and suicide; studies also considered epigenetic modification of a diverse array of other genes.

Given the small number of studies, drawing substantive conclusions about how epigenetic modifications in specific genes may be operating in the etiology of the diseases in question is not possible at this stage. Our review occasions a synthesis of methodological limitations of the extant literature and recommendations on how investigators may best approach this area in future studies.

Five methodological limitations to this literature emerge from our review. The first is that studies in this area have suffered from small sample sizes, the consequences of which include lack of power and increased false discovery rates. Second, existing studies have been limited to assessing epigenetic modification in the post-mortem brain or the peripheral blood following disease diagnosis, and drawing inference from either tissue type is problematic. Third, studies have used different techniques to assess epigenetic modifications that may produce heterogeneous results. Fourth, few studies have assessed environmental antecedents to epigenetic modifications in extant studies. Fifth, there appears to be little consensus regarding genome-wide vs. candidate-gene approaches.

The first methodological limitation to this literature is the use of small sample sizes in most studies, a ubiquitous problem in molecular epidemiology [33]. Of the studies we reviewed, only one included more than fifty cases (e.g., subjects with the outcome). Compounding small sample sizes in studies overall, many of the studies we reviewed limited epigenetic analysis to a subset of the total study population. Small sample sizes limit study power, therefore increasing the likelihood of type II error (e.g., the proportion of false negative findings) [33]. More dangerously, underpowered studies also increase the “false discovery rate” or the number of significant findings that fall into type I error (e.g., the proportion of false positive findings), as demonstrated in Equation 1 [34-36].

(1) $$FDR=\frac{\alpha \left(1-prior\right)}{\alpha \left(1-prior\right)+power*prior}$$

^ “prior” indicates the proportion of tested hypotheses that are actually correct.

In this equation, the false discovery rate (FDR) is inversely proportional to power (1-beta), such that low power also yields high FDR, driving up Type I error. Therefore, given the small sample sizes employed in the majority of studies we reviewed, it is likely that the findings suffer from high proportions of both type I and type II error.

A second limitation is the use post-mortem brain or peripheral cell tissues for epigenetic analyses. Seven of the 21 studies we reviewed analyzed epigenetic modification in peripheral blood cells, and one study analyzed epigenetic modification in buccal mucosa. Although all human cells carry the full endowment of genetic material, cells modify gene expression to efficiently carry out their diverse functions as they specialize, silencing some genes while activating others in line with their physiologic responsibilities. Epigenetic modification is the physiologic process by which genes are silenced or primed for expression [37,38]. The pathophysiology of mood-anxiety disorders and suicide is localized to the brain and it remains therefore unclear how gene expression in peripheral tissues correlates with physiologically meaningful gene expression in the brain. However, even epigenetic studies using post-mortem brain tissue have challenges. Three of the 21 studies we reviewed analyzed post-mortem brain tissue. While these studies assessed epigenetic changes in the appropriate organ, assessing post-mortem brain tissue carries its own challenges. This is problematic with respect to temporality between exposure and outcome, because post-mortem brains, by definition, can only be harvested after death, and therefore epigenetic modification can only be ascertained after the occurrence of the outcome. Moreover, death often involves acidosis, which may contribute to the instability of genetic material [39-41], increasing the likelihood of misclassifying epigenetic modification and increasing the chances of spurious findings. Therefore, much more work is needed to help us understand the physiologic significance of both peripheral tissue and brain methylation patterns.

A third limitation to the literature is that published studies used different laboratory techniques to measure the degree of epigenetic modification. With respect to DNA methylation alone, there are a number of gene-specific assays currently in use, including bisulfite reaction based DNA sequencing methods, which include bisulfite genomic sequencing PCR [42] and/or methylation specific PCR [43]; genome-wide screens, such as CpG island microarrays [44] and Restriction Landmark Genomic Scanning for Methylation (RLGS-M) techniques [45]; and methylated DNA immunoprecipitation (MeDIP) [46]. There are few studies that have compared the sensitivity and specificity of each method, although a recent study compared two bisulfite sequence-based assays (which are very similar) head-to-head and found as much as an 18% difference in identification of methylated CpG islands in biological replicates of human embryonic stem cells [47]. To our knowledge, there are no “gold standard” assays for most epigenetic markers. Therefore, differential use of assays may present a source of misclassification bias in studies, which would ultimately increase the rate of type II error rate in the extant literature.

Fourth, studies in our review largely failed to assess the environmental exposures thought to produce epigenetic change to begin with. Only three out of 21 of the studies reviewed here included any assessment of a common environmental stressor with respect to epigenetic modifications and their relationship with common mood and anxiety disorders and suicide [14,19,20]. This is an important limitation, as there is ample data demonstrating the importance of environmental stressors in the etiology of these disorders [48]. Without assessing common environmental stressors antecedent to epigenetic modifications, our studies fail to adequately test dominant hypotheses about the mediating role of epigenetic changes between environments and outcomes in common mood and anxiety disorders and suicide.

The fifth limitation is the lack of consensus regarding genome-wide vs. candidate-gene approaches in epigenetic studies. Three of the studies we reviewed used genome-wide approaches [12,30,32], while the remaining 18 studies assessed for epigenetic modifications of candidate genes. Both approaches have limitations. With respect to genome-wide studies, the analyses (and findings) are often unfocused. Unlike in genome-wide association studies, there is no agreed upon method for analysis and synthesis of data or for adjustment for multiple comparisons in genome-wide epigenetic studies. In particular, appropriate adjustment for multiple comparisons can be problematic, increasing the proportion of false-positive findings [49]. Candidate-gene approaches benefit from being hypothesis-driven, and therefore more amenable to thoughtful, model-based study designs. However, candidate-gene approaches face their own limitations. Candidate-gene studies are more likely to yield overall negative results, as these studies test only one hypothesis, as compared to genome-wide studies which test more global hypotheses about the role of epigenetic modification *anywhere* in the genome influencing risk for outcomes of interest. As candidate-gene approaches are more likely to yield negative overall findings, there is a high probability of publication bias, whereby the literature about candidate-gene epigenetic modification is likely to be highly enriched for positive findings [35].

### Limitations of the review

The reader should be aware of several limitations when considering the findings of our systematic review. First, we limited our review to the published peer-reviewed literature. Therefore, it is plausible that our selection of studies may have been subject to a publication bias, affecting the veracity of our inferences. Second, we organized studies by outcome. This organizational scheme may have also, in part, shaped the inferences drawn here. However, because we limited our inferences largely to methodological critiques of the literature, it is unlikely that either limitation would have had a substantial influence on our interpretation of our findings. Third, our review was limited to only the English-language literature published in journals indexed in two databases. It is plausible that we may have missed literature about epigenetics in relation to common mood and anxiety disorders and suicide published in other languages or in journals that were not indexed in MEDLINE or PSYCHINFO. However, this is less likely, as a detailed search of the citations of included studies yielded no further studies for inclusion in the review.

## Conclusions

Research into the epigenetic mechanisms that may underlie common mood and anxiety disorders and suicide has the potential to unite the heretofore disparate bodies of work that have characterized the pathophysiology of these disorders and their population causes, respectively. However, at this nascent stage in the development of this literature, there are several methodological challenges, discussed above, that have yet to be addressed. With respect to these challenges moving forward, studies about the role of epigenetic regulation in the etiology of common mood and anxiety disorders can improve in the following ways.

As sufficient power and small sizes have dogged extant studies in this area, future studies require larger sample sizes, maximizing power and minimizing the false discovery rate. Furthermore, systematic comparisons of assays used to assess epigenetic modifications are needed as is consensus around what constitute ‘gold standard’ laboratory techniques for assessing epigenetic modification. Another challenge to this literature is the validity of using epigenetic data from peripheral blood, epithelial tissue, or post-mortem brain specimens. However, given the obvious limitations to sampling brain tissue in representative populations of living subjects, sampling these tissues may, in the long-term, remain the best available option. Future work associating epigenetic modifications with changes in gene expression and correlating epigenetic modifications in peripheral tissues with brain function may offer one way to address these limitations. Moreover, work in neuroscience to characterize the relationship between real-time brain imaging studies and epigenetic modification in brain regions sampled among patients undergoing neurosurgery, when living-patient brain samples can be collected, could yield imaging markers of epigenetic regulation in the brain. These approaches represent a handful of the many lines of inquiry that could improve our capacity to assess epigenetic modification in the brain. Future studies in this area would also do well to measure epigenetic modification in relation to its environmental antecedents so as to better assess dominant hypotheses about the mediation of the relationship between environmental exposures and common mood and anxiety disorders and suicide by epigenetic modification.

Lastly, both genome-wide and candidate gene approaches have their role in epigenetic analyses. Genome-wide approaches may be more appropriate for exploratory analyses [50]. However, candidate gene approaches may be better suited for hypothesis-testing regarding the roles of individual genes or sets of genes hypothesized to function in a particular way in disease etiology. It is important that investigators in this area are attuned to the particular strengths and weakness of each approach so that each is used appropriately in studies about the role of epigenetics in the etiology of common mood and anxiety disorders and suicide.

## Competing interests

The authors declare that they have no competing interests.

## Authors’ contributions

AME extracted data from studies and drafted the manuscript. MRH carried out the literature search and edited the manuscript for intellectual content. SG edited the manuscript for intellectual content. KCK conceived the review, specified review inclusion and exclusion criteria, and edited the manuscript for intellectual content. All authors have read and approve the final version.

## Author details

^1^Department of Epidemiology, Mailman School of Public Health, Columbia University, 722 W. 168th Street, R521, New York, NY 10032, USA. ^2^College of Physicians and Surgeons, Columbia University, New York, NY, USA.^3^Department of Sociomedical Sciences, Mailman School of Public Health, Columbia University, 722 W. 168th Street, New York, NY 10032, USA.^4^Department of Epidemiology, Mailman School of Public Health, Columbia University, 722 W. 168th Street, 1508, New York, NY 10032, USA. ^5^Department of Epidemiology, Mailman School of Public Health, Columbia University, 722 W. 168th Street, R720G, New York, NY 10032, USA.
